# Complete plastome sequence of *Limonium aureum,* a medicinal and ornamental species in China

**DOI:** 10.1080/23802359.2019.1703608

**Published:** 2019-12-18

**Authors:** Xiaoyin Zhang, Yao Xu, Xiao Liu

**Affiliations:** Key Laboratory of Resource Biology and Biotechnology in Western China, Ministry of Education, Northwest University, Xi’an, China

**Keywords:** *Limonium aureum*, chloroplast genome, phylogenetic analysis

## Abstract

*Limonium aureum* is a perennial herb of Plumbaginaceae, and draw the attention of researchers by its medicinal and ecological value. In this study, we first report the complete chloroplast genome of *L. aureum* with paired-end sequencing method. The results showed that the complete chloroplast genome of *L. aureum* is 154,661 bp in length with a typical quadripartite structure, including a large single-copy region (LSC, 84,545 bp), a single-copy region (SSC, 12, 980 bp), and a pair of inverted repeats (IRs, 28,568 bp). There are 113 annotated genes, consisting of 79 unique protein-coding genes, 4 unique ribosomal RNA genes, and 30 transfer RNA genes. Moreover, we constructed a phylogenetic tree with *L. aureum* and other 34 species based on their complete chloroplast genomes. And the results of the phylogenetic topologies displayed that *Plumbago auriculata* was closely related to *L. aureum.* Our results will contribute to the better study and make use of the species.

*Limonium aureum* (L.) Hill is a perennial herb, belonging to Plumbaginaceae family, mainly distribute in the northeast and northwest provinces of China, it has also been found in Ganzi region in Sichuan province in recent years (Tse-hsiang Peng 1987). *L. aureum* attracts many researchers’ attention for its medicinal, ornamental, and ecological values (Ye and Huang 2006). Previous studies mainly focused on the morphological and physiological aspects of *L. aureum* (Li et al. 2007; Geng et al. 2015; Liu et al. 2016). The investigation on the molecular data of this species will contribute to the better study and utilization. In this study, we would first report the chloroplast genome of *L. aureum* based on paired-end sequencing.

Fresh leaves of *L. aureum* were sampled from the Yinchuan Botanical Garden (38°28′N, 106°16′E; Ningxia, NW China). A voucher specimen (TB121612) is deposited at the herbarium, the Department of Life Sciences in Xi’an Northwest University. We used the modified CTAB method to extract the total genomic DNA (Doyle and Doyle, 1987). A shotgun library constructed following the manufacturer’s protocol for the Illumina HiSeq X Ten Sequencing System (Illumina, CA, USA). We assembled cp genome using the program MITObim v1.8 (Hahn et al. 2013), with that of *Plumbago auriculata* (GenBank: *MH286308*) as the initial reference. The genome was annotated using software Geneious v9.0.2 (Biomatters Ltd., Auckland, New Zealand) by aligning with the reference chloroplast genome. The circular plastid genome map was completed using the online program OGDRAW (Lohse et al. 2013). The annotated chloroplast genome sequence has been deposited into the GenBank with the accession number *MN623109*.

The results showed that plastome of *L. aureum* possess a total length 154,661 bp, containing two inverted repeats (IRs) of 28,568 bp, a large single-copy (LSC) region of 84,545 bp, and a small single-copy (SSC) region of 12,980 bp. The plastome contains 113 genes, consisting of 79 unique protein-coding genes, 30 unique tRNA genes, and 4 unique rRNA genes. The overall A/T content in the plastome is 63.00%, for which the corresponding value of the LSC, SSC, and IR regions were 64.70%, 68.50%, and 59.20% respectively. In addition, 9 PCG genes (*atpF, ndhA, ndhB, petB, petD, rpl2, rps12, rps16* and *rpoC1*) possess a single intron, 96 PCG genes no intron, 2 other genes (*clpP* and *ycf3*) harbor two introns. 6 tRNA genes (trnA-UGC, trnG-UCC, trnK-UUU, trnL-UAA, trnI-GAU and trnV-UAC) harbor a single intron.

Furthermore, to investigate the phylogenetic position of *L. aureum*, The neighbor-joining (NJ) phylogenetic tree was constructed (Figure 1) based on 35 complete chloroplast genome sequences from published species using MEGA7 with 1000 bootstrap replicates (Kumar et al. 2016). According to the phylogenetic topologies, *L. aureum* was closely related to *Plumbago auriculata*. The complete plastome sequence of *L. aureum* will not only provide effective use of this species, but also for the phylogenetic studies of Plumbaginaceae.

**Figure 1.**
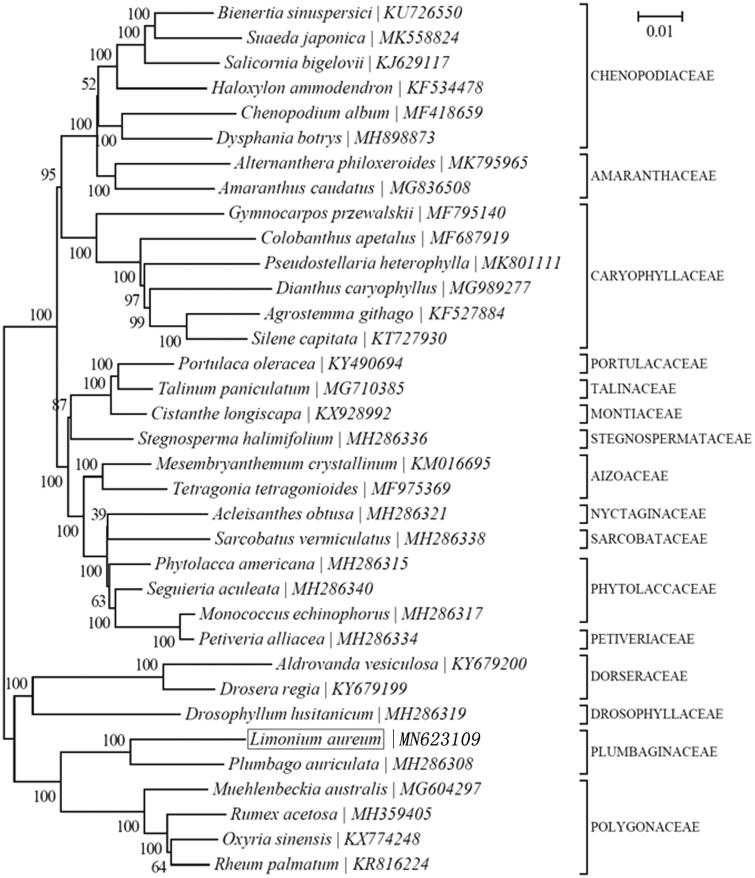
Neighbor-joining (NJ) phylogenetic tree based on 35 complete chloroplast genomes using MEGA7 with 1000 bootstrap replicates.
